# Transrectal Ultrasound and Photoacoustic Imaging Probe for Diagnosis of Prostate Cancer

**DOI:** 10.3390/s21041217

**Published:** 2021-02-09

**Authors:** Jihun Jang, Jinwoo Kim, Hak Jong Lee, Jin Ho Chang

**Affiliations:** 1 Department of Electronic Engineering, Sogang University, Seoul 04107, Korea; jhjang@sogang.ac.kr; 2 Department of Information and Communnication Engineering, Daegu Gyeongbuk Institute of Science and Technology, Daegu 42988, Korea; kimjw0319@dgist.ac.kr; 3 Department of Radiology, Seoul National University of Bundang Hospital, Seongnam-si 13620, Korea; hakjlee@snu.ac.kr

**Keywords:** transrectal probe, optical lens, ultrasound imaging, photoacoustic imaging, prostate cancer

## Abstract

A combined transrectal ultrasound and photoacoustic (TRUS–PA) imaging probe was developed for the clear visualization of morphological changes and microvasculature distribution in the prostate, as this is required for accurate diagnosis and biopsy. The probe consisted of a miniaturized 128-element 7 MHz convex array transducer with 134.5° field-of-view (FOV), a bifurcated optical fiber bundle, and two optical lenses. The design goal was to make the size of the TRUS–PA probe similar to that of general TRUS probes (i.e., about 20 mm), for the convenience of the patients. New flexible printed circuit board (FPCB), acoustic structure, and optical lens were developed to meet the requirement of the probe size, as well as to realize a high-performance TRUS–PA probe. In visual assessment, the PA signals obtained with the optical lens were 2.98 times higher than those without the lens. Moreover, the in vivo experiment with the xenograft BALB/c (Albino, Immunodeficient Inbred Strain) mouse model showed that TRUS–PA probe was able to acquire the entire PA image of the mouse tight behind the porcine intestine about 25 mm depth. From the ex vivo and in vivo experimental results, it can be concluded that the developed TRUS–PA probe is capable of improving PA image quality, even though the TRUS–PA probe has a cross-section size and an FOV comparable to those of general TRUS probes.

## 1. Introduction

Transrectal ultrasound (TRUS) has been used for the screening and diagnosis of prostate cancer, which is one of the most common cancers occurring in adult men [1]. For imaging, a TRUS probe is inserted into the rectum. Therefore, it is desirable that the size of TRUS probes should be as small as possible, to relieve of the patient’s pain during imaging. In order to image the entire prostate, the field-of-view (FOV) of conventional TRUS probes should be as large as possible. These two restrictions limit the spatial and contrast resolutions of TRUS images, because aperture size is one factor determining the spatial resolution and signal-to-noise ratio of ultrasound (US) images, and physical conformation for wide FOV possibly degrades the sensitivity of US probes. For these reasons, TRUS imaging does not provide enough resolution and sensitivity to clearly identify and locate prostate cancers (especially early stage prostate cancers) and to accurately distinguish prostate cancers from benign prostatic hyperplasia [2]. In addition, the accuracy of TRUS-image-guided biopsy is only 20 to 30% [3], because optimal biopsy sites are not clearly shown on TRUS images, thus requiring repeated biopsies at the expense of the cost of diagnosis and the risk of complications.

Contrast-enhanced ultrasound (CEUS), in combination with TRUS imaging, has been used successfully to improve diagnostic accuracy of prostate cancer [4,5,6]. Since CEUS facilitates clear visualization of micro- and neo-vascularization, the success rate of TRUS image-guided biopsy is increased. This is because hyper-vascularity is typically observed in the periphery of prostate cancer [5,7]. However, CEUS is less sensitive to small blood vessels and slow blood flow, even if US contrast agents are used [6]. Note that it is considered that those are the indicators associated with early stage cancers [8].

On the other hand, photoacoustic (PA) imaging is highly sensitive to blood vessels [9] and biopsy needles [10]. Therefore, combined TRUS and PA (TRUS–PA) imaging can be a solution to the problems of CEUS and TRUS imaging if high performance TRUS–PA probes are available. The first feasible study was conducted to demonstrate that TRUS–PA imaging can be used for accurate diagnosis of prostate cancer due to the clear visualization of microvasculature distribution in the prostate [11,12]. For the pilot study, a TRUS–PA probe with a wide FOV of 160° was developed; it consisted of a 128-element, 6.5 MHz TRUS array transducer and two convex-shaped optical modules for irradiated light to cover the wide FOV [11]. Note that no detailed technical information about the specifications of the TRUS transducer, the design of the optical modules, the integration of the TRUS, and optical modules could be found. As another approach, it has recently been reported that a 64-element, 5 MHz linear capacitive micromachined ultrasonic transducer (CMUT) could be used for TRUS–PA imaging [13]. The CMUT array was a side-looking transducer with a FOV of 40°, and three optical fiber bundles were placed on three sides of the CMUT array, to create dark field light illumination. However, the CMUT-based TRUS–PA probe should be further improved, because general TRUS probes are forward-looking transducers and have a wide FOV larger than 130°, to ensure diagnostic efficiency. In addition, both types of the TRUS–PA probes have a maximum cross-sectional size of 25 mm or more, and that is larger than general TRUS probes.

Since a TRUS transducer should be integrated with an optical module for TRUS–PA imaging of the prostate, it is challenging that the TRUS–PA probe is similar in size to general TRUS probes but has a large FOV. In this paper, we report a recently developed TRUS–PA probe that meets both requirements of size and FOV; the objective of the development was that the TRUS–PA had a size and FOV similar to conventional TRUS probes, with which high-quality PA images could be obtained. To achieve the development goal, particularly, the optical lens was designed to have a concave–convex shape in the lateral-axial plane for divergence and a planar–oblique shape in the elevation-axial plane for refraction. The TRUS–PA probe developed here consisted of a miniaturized 128-element 7 MHz convex array transducer with a FOV of 134.5°, a bifurcated optical fiber bundle, and two optical lenses; the maximum cross-sectional size of the TRUS–PA probe was about 20.5 mm, which is similar to that of the commercial TRUS transducers. From ex vivo and in vivo experiments, it was ascertained that the developed optical lens facilitates efficient delivery of light to the imaging plane (i.e., lateral-axial plane). In this study, additionally, light penetration through the porcine intestine was measured as a function of wavelength, to determine an optimal wavelength for PA imaging of the prostate. This was necessary because radiated light should penetrate the wall of the rectum, to reach the prostate, and it is known that light absorption highly occurs in the rectal wall.

## 2. Transrectal Ultrasound and Photoacoustic Imaging Probe

**Figure 1:**
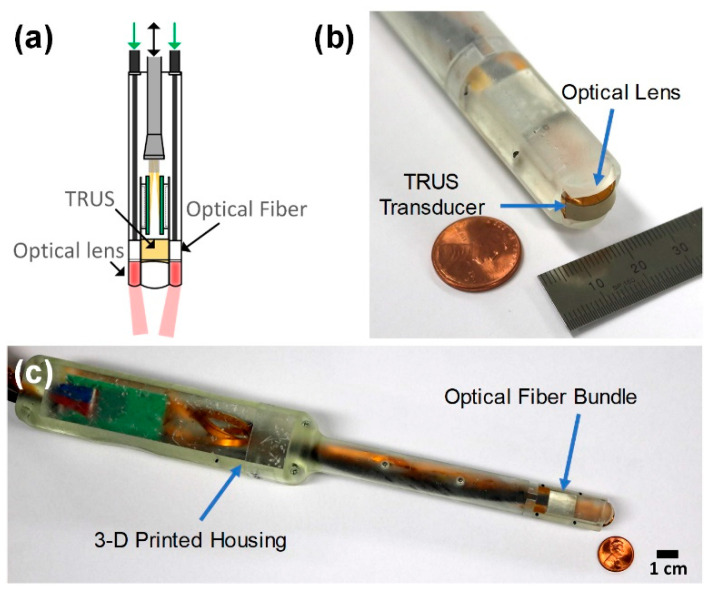
(**a**) Schematic of the developed transrectal ultrasound–photoacoustic (TRUS–PA) probe consisting of a TRUS array transducer, two optical fiber bundles, and two optical lenses. (**b**,**c**) Photographs of the developed TRUS–PA probe.

The developed TRUS–PA probe consists of an optical module, a TRUS array transducer, and a housing. The goal in developing the TRUS–PA probe was to make its diameter similar to that of general TRUS probes (i.e., about 20 mm), for the convenience of the patients. Note that the patients generally complain of great pain when a TRUS probe is inserted into the rectum for prostate imaging; the smaller the probe size, the better. Moreover, the probe was designed to have an imaging plane covering the prostate gland volume that typically measures 30 mm (anteroposterior) × 30 mm (width) × 50 mm (longitudinal) [7,14]. The goal could be achieved by developing a miniaturized convex ultrasound array and an optical lens, as shown in Figure 1; the cross-sectional size of the front part of the developed TRUS–PA probe was 14 mm × 15 mm. To the best of our knowledge, this size is the smallest of the reported TRUS–PA probes. Each component of the developed TRUS–PA is described here.

### 2.1. Optical Module

**Figure 2:**
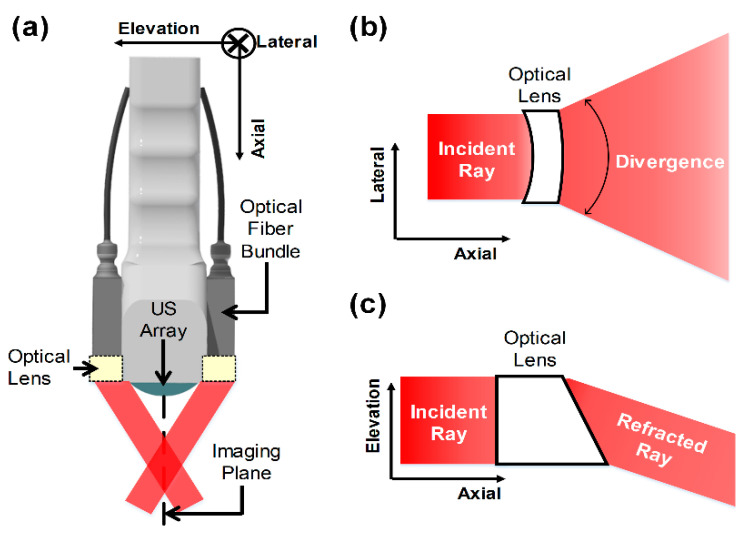
Conceptual illustration of (**a**) the developed TRUS–PA probe with two optical lenses placed on each side of the ultrasound (US) array transducer and the desired optical lens with (**b**) a concave–convex shape in the lateral-axial plane and (**c**) a planar–oblique shape in the elevation-axial plane.

For high-quality PA images, light should be delivered to an imaging plane efficiently (see Figure 2). A simple and general way is to place two optical fiber bundles on each side of an US transducer and to tilt the fiber bundles at a certain angle, so that the beams overlap at the desired depth in an imaging plane [15,16,17]. As another way, optical reflectors attached to one side of an US transducer can be used to deliver light to the imaging plane [18,19]. However, these methods inevitably result in increasing the size of a US–PA probe, thus being not suitable for a TRUS–PA probe. For the sake of small size, optical fiber bundles can be simply attached parallel to each side of an US transducer. If the outlets of optical fiber bundles have a large numerical aperture, emitted light can spread at a large angle, so that the light can cover the region of interest (ROI) in the desired imaging plane. Although the divergent beam may be a feasible solution for a TRUS–PA probe with small external size and large FOV, the light fluence delivered in ROI is too small to be suitable for high-quality PA imaging. This is because emitted light can suitably spread in the lateral-axial plane, but much of the light cannot reach an imaging plane, due to no focusing on the elevation-axial plane. Note that PA signal intensity is linearly proportional to light fluence. Additionally, undesired PA signals are possibly generated from the off-axis of an imaging plane and received by a US transducer, thus degrading PA image quality.

For large FOV and light focus on ROI, while minimizing the size of the TRUS–PA probe, we designed an optical lens, as shown in Figure 2; the desired optical lens should produce a divergent beam in the lateral-axial plane that is equal to the imaging plane (Figure 2b) and a refracted beam in the elevation-axial plane (Figure 2c). To obtain the properties, the optical lens should have a concave–convex shape in the lateral-axial plane for divergence and a planar–oblique shape in the elevation-axial plane for refraction. The optical lens was designed, using the ray-tracing technique [20,21], to determine key parameters for fabrication of the lens: radius of curvature of the concave and convex boundaries for the concave–convex lens, and inclination angle of the oblique boundary for the planar–oblique lens. For the sake of simplifying the design, we assumed that a ray was a collimated beam (i.e., light diffraction was not considered) and ignored the law of reflection. Since the output aperture of the optical fiber bundle used for this study was configured as a 13 mm × 2 mm rectangle, the width of the collimated beam was set to be 13 mm in the lateral-axial plane and 2 mm in the elevation-axial plane. Therefore, the lens thickness in the elevation direction was selected to be 2 mm. Note that the custom-made optical fiber bundle had a numerical aperture (NA) of 0.22, so that the emitted light could be approximately considered as a collimated beam. The focal length of the lens was determined to be 25 mm, considering the longitudinal size of the prostate. Note that most prostate cancers occur at a depth of less than 30 mm from the rectal wall [22]. As a lens material, we selected Epotek-301 (Epoxy Technologies, Billerica, MA, USA), because the optical transparency of the material is 0.95 in the 382–1640 nm range [23]; its refractive index is 1.519. The equations derived for the optical lens design based on the ray-tracing method and numerical-simulation results can be found in Appendix A.

**Figure 3:**
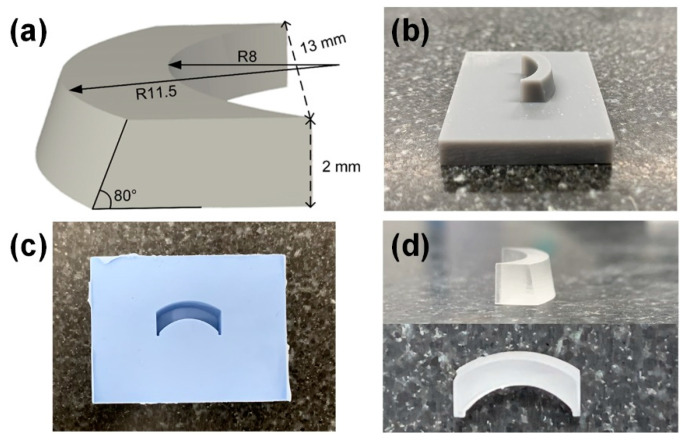
(**a**) Optical lens designed by a 3D CAD (Computer Aided Design) program; (**b**) positive optical lens mold constructed by using a 3D printer; (**c**) negative optical lens mold constructed by using Room-Temperature Vulcanizing (RTV), to fabricate the optical lens with the lens material, i.e., Epotek-301; and (**d**) completed optical lens of which inner and outer radii were 8 and 11.5 mm, respectively, and oblique angle was 80°.

As a result, the concave–convex radii were selected to be 8 and 11.5 mm (see Figure 3a). In this case, an FOV of 105° in the lateral-axial plane was expected. Moreover, the inclination angle of the oblique boundary for the planar–oblique lens was determined to be 80°. With these parameters, a positive mold for the optical lens was designed by using a 3D CAD (Computer Aided Design) program and created by using a 3D printer (Form 2, Formlabs, MA, USA), as shown in Figure 3a,b. Glass plates surrounded the positive mold, to construct dams, and Room-Temperature-Vulcanizing (RTV) silicone rubber (RTV664, Momentive Performance Material Inc., Waterford, NY, USA) was poured into the positive mold and cured at room temperature, for 24 h. The negative RTV mold was prepared after removing the positive mold (Figure 3c). Epotek-301 resin and hardener were mixed at a ratio of 4:1, and the epoxy mixture was degassed for 10 min. The epoxy mixture was poured into the negative RTV mold and cured at room temperature, overnight, in a dry box. Finally, the completed optical lens was separated from the RTV mold, as shown in Figure 3d.

### 2.2. Transrectal Ultrasound Array

**Figure 4:**
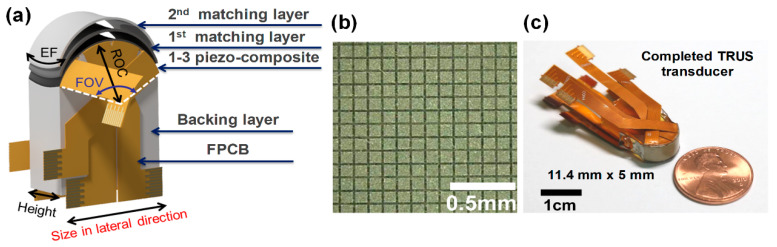
(**a**) Description of acoustic structure of the developed TRUS probe, (**b**) photograph of the finished 1–3 piezocomposite, and (**c**) photograph of the finished TRUS probe. Reprinted with modification from Reference [24].

We designed and fabricated a miniaturized 7 MHz TRUS array transducer, of which the footprint was 11.4 mm (lateral) × 5 mm (elevation): 128 elements, 30 mm elevational focal length, and 134.5° FOV. Geometric focus in the elevation direction, instead of lens focus, was employed to avoid ultrasound attenuation in an acoustic lens material. Therefore, the array transducer had a saddle-shaped aperture (Figure 4a). The first and second acoustic matching layers were 2–3.5 μm silver-loaded epoxy and mixture of Insulcast 502 and Insulcure 9. The backing block was constructed by using Epotek-301. To obtain high transmission and reception efficiency, additionally, a PZT-5H-based 1–3 piezocomposite was designed and fabricated (Figure 4b). For a small-sized TRUS probe, FPCB (flexible printed circuit board) should be completely bent perpendicular to the convex surface. For this, a new structure of FPCB, with several strain relief slits between the signal trace groups, was developed (Figure 4c). The center frequency and −6 dB fractional bandwidth of the fabricated TRUS array were measured at 6.75 MHz and 66%, respectively. The detailed fabrication process and imaging performance of the TRUS array developed for the TRUS–PA probe can be found in Reference [24].

### 2.3. Housing

**Figure 5:**
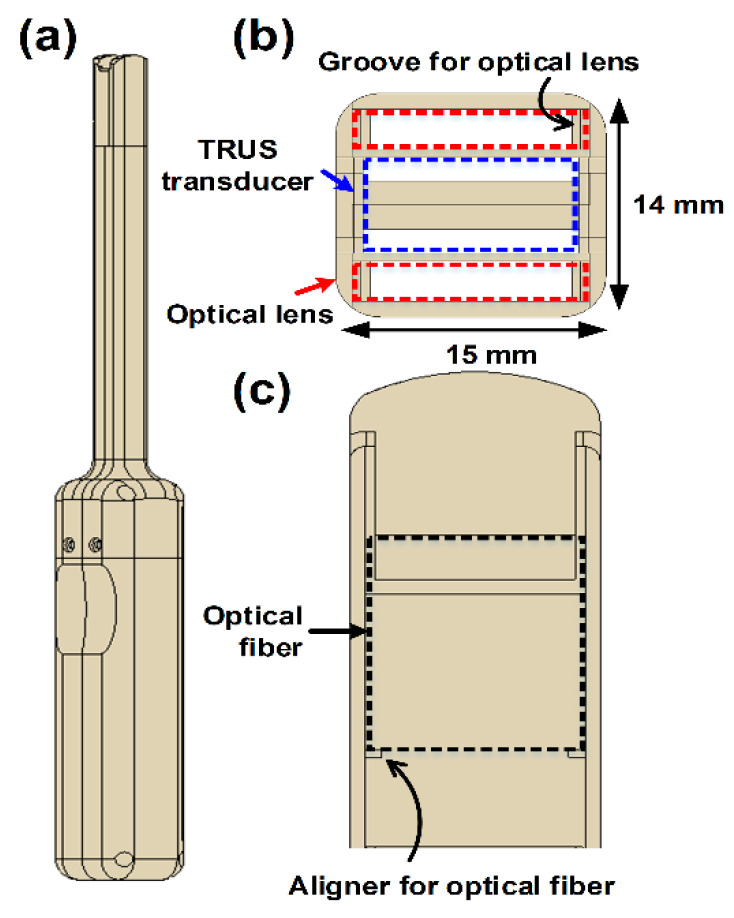
(**a**) Schematic illustration of the custom-designed housing of the TRUS–PA probe: (**a**) side view and (**b**) front view of the housing, and (**c**) cross-section view of the housing tip.

The TRUS–PA probe housing was designed using a 3D CAD software to integrate the miniaturized TRUS array, optical lens, and bifurcated optical fiber bundles (Figure 5). The cross-section size of the front part of the housing, that is inserted into the patient’s rectum for imaging, was determined to be 14 mm × 15 mm, considering the usefulness in the diagnosis and the alleviation of the patient’s pain during imaging (Figure 5b). The housing had two grooves for mounting the fabricated optical lenses. Moreover, the outlets of the optical fiber bundles were fixed on the aligners in the housing (Figure 5c). A prototype of the TRUS–PA probe housing was constructed using the 3D printer, and the material of the housing was biocompatible photopolymer resin. Figure 1 shows the photographs of the completed TRUS–PA probe. The remarkable fact is that the maximum cross-sectional size of the developed TRUS–PA probe was about 20.5 mm, which was comparable to the commercial TRUS transducers although the probe contained both acoustic and optical modules.

## 3. Performance Evaluation and Discussion

### 3.1. Light-Intensity Distribution

**Figure 6:**
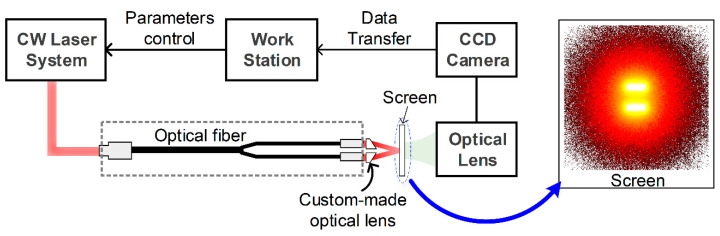
Illustration of the experimental setup for light-intensity distribution measurement as a function of depth. CW, continuous wave; CCD, charge-coupled device.

The performance of the developed optical lens was evaluated by measuring the light-intensity distribution as a function of depth. A continuous wave (CW) laser system (Nova Pro., RGB Photonics GmbH, Kelheim, Germany) was used to deliver a CW laser with a wavelength of 520 nm to a custom-made bifurcated optical fiber bundle with an NA of 0.66 (see Figure 6). Since irradiated light is scattered in biological media, we selected the fiber bundle with a relatively large NA; otherwise, the light hardly reached an imaging plane without the developed optical lens when the outlets of the fiber bundle were parallel to light propagation direction. For evaluating the performance of the optical lenses, we placed the optical lenses as close as possible to the bundle outlets, because it was assumed that a collimated beam entered the optical lens. Light intensity was measured after an optical screen was placed at a desired distance from the outlets of the fiber bundle, i.e., 10 to 60 in 10 mm increments. The light-intensity distribution on the screen was detected and recorded, using a charge-coupled device (CCD) camera (CoolSNAP MYO, Photometrics, Tucson, AZ, USA) equipped with an optical lens (Micro-Nikkor 105 f/2.8, Inc., Rochester, NY, USA).

**Figure 7:**
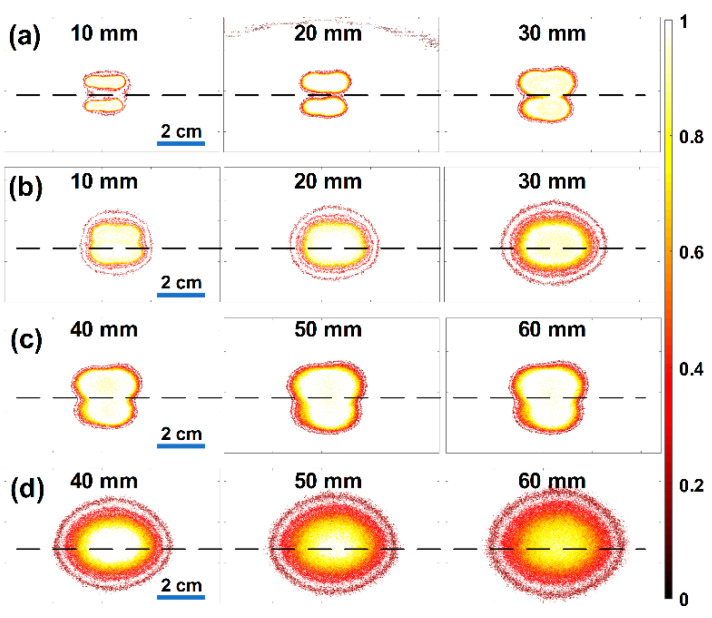
Measured light-intensity distributions at depths of 10 to 60 mm in 10 mm increments: (**a**,**c**) without the optical lenses and (**b**,**d**) with the optical lenses. The black dashed lines on each image indicate the imaging plane that is the center position of the US array transducer.

**Figure 8:**
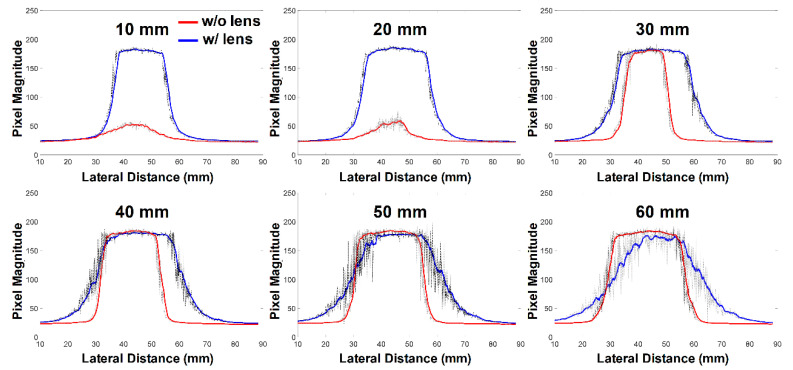
Light-intensity profiles measured along horizontal axis (i.e., imaging depth), indicated by the black dashed lines in Figure 7. The red and blue solid lines represent the moving averaged light intensities, without and with the optical lenses, respectively. The black lines indicate the measurement data.

As shown in Figure 7a,c, the light-intensity distribution without the optical lens was naturally diffused, because an uncollimated beam (i.e., an NA of 0.66) was irradiated. The diffusion in biological media may be beneficial to a small-sized TRUS–PA probe in which optical fiber bundles are simply attached parallel to each side of an US transducer. In this particular experiment, the light beams irradiated from two optical fiber bundle outlets were separated from one another at depths of 10 and 20 mm, and these began to overlap after a depth of 30 mm (see Figure 7a). Since the irradiated light beams did not overlap completely in the imaging plane, the light intensity was weak in the imaging plane (i.e., lateral-axial plane), and the FOV of PA images was predicted to be narrow, as shown in the top panel of Figure 8. In this depth, additionally, the light intensity was strong in the off-axis of an imaging plane, thus resulting in reducing spatial and contrast resolutions of PA images; the adverse effect occurs for a similar reason that the spatial and contrast resolutions of US images are reduced due to large slice thickness (i.e., elevation resolution) [25]. In contrast, the light beams passing through the optical lenses overlapped from a depth of 10 mm (Figure 7b), and the light intensity at depths of 10 and 20 mm was about 5.3 and 4.6 times higher than that of the light delivered without the optical lens (the top panel of Figure 8). Additionally, the light-intensity distribution was wider in the imaging plane when the optical lens was used. This is because the lens had the ability to spread the irradiated light in the lateral-axial plane and refract it in the elevation-axial plane. The full-width at half maximum (FWHM) of the irradiated light through the lens was 20.2, 25.5, and 28.9 mm at depths of 10, 20, and 30 mm, whereas that of the light without the lens was 17.4, 12.3, and 15.8 mm. The maximum intensity of the light through the lens was similar to that without the lens at a depth of 30 mm as shown in Figure 8. After this depth, the maximum intensity of the light through the lens decreased slightly with depth, because the focal length of the planar–oblique lens in the elevation-axial plane was 25 mm and the light continued to spread in the lateral-axial plane; however, the FWHM also continued to broaden, i.e., 31.1, 32.1, and 32.5 mm at depths of 40, 50, and 60 mm, whereas the FWHM of the light irradiated without the lens was 21.8, 25.0, and 27.8 mm at depths of 40, 50, and 60 mm (the bottom panel of Figure 8). Note that moving averaging filtering with a length of 30 was performed for smoothing the pixel data indicated by the black lines.

**Figure A3:**
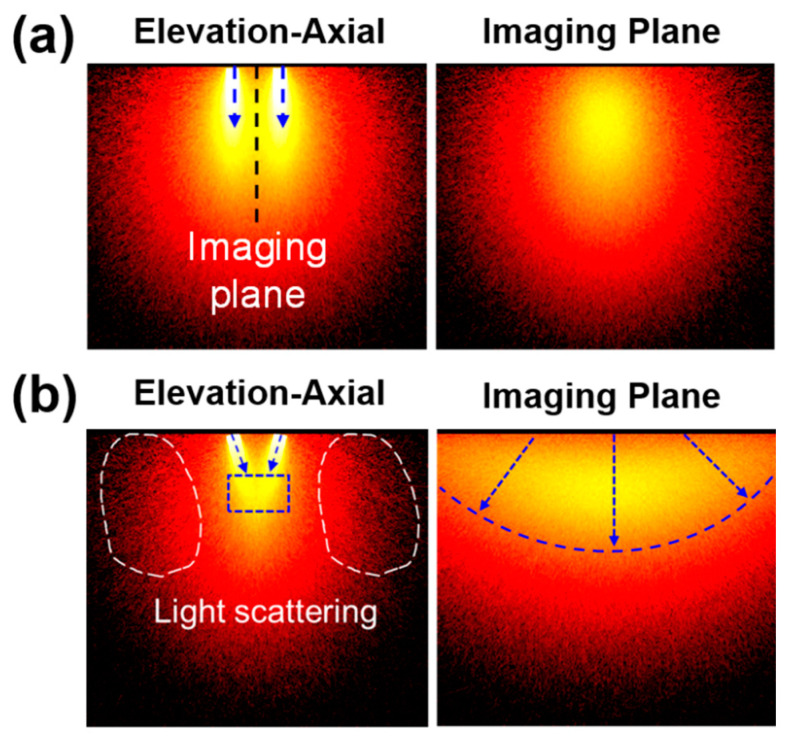
Results of the Monte Carlo simulation for light propagation in optical scattering media; light is delivered (**a**) in parallel and (**b**) at an oblique angle.

The experimental results implied that the developed optical lens was predicted to be beneficial for PA image quality improvement and wide FOV. However, the performance may be different in biological media in which irradiated light spreads rapidly due to optical scattering, depending on the type of biological media [26]. In the results of Monte Carlo simulation (see Appendix C Figure A3), it was observed that the direction of the light scattering is dominated by the energy distribution of the initially irradiated light. Therefore, the developed optical lens was also expected to play an important role in increasing FOV and improving PA image quality in biological media. This was confirmed through the following experiments conducted to evaluate imaging performance.

### 3.2. Imaging Performance

**Figure 9:**
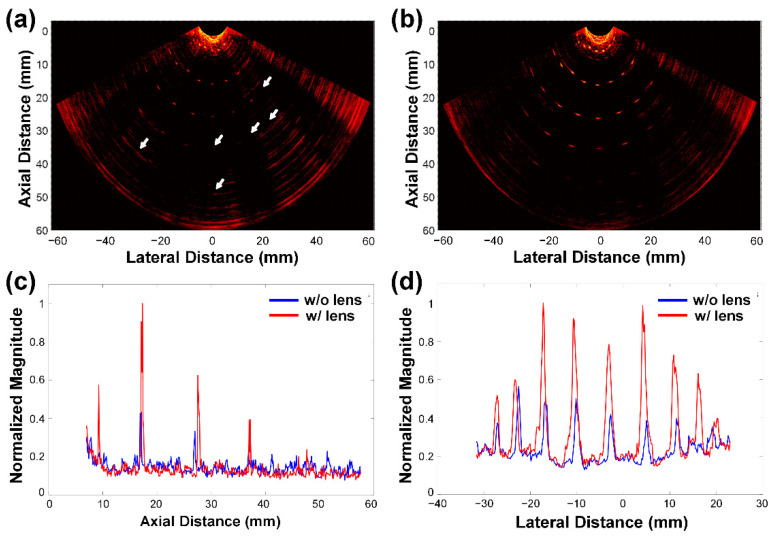
PA images of tungsten wire targets, (**a**) without and (**b**) with the developed optical lens. The white arrows in (**a**) indicate the invisible wire targets on the PA image in (**a**), but visible in (**b**). (**c**,**d**) Normalized envelope profiles of the wire target images along (**c**) the axial direction at the center position in the lateral direction and (**d**) the lateral direction at a depth of 28 mm.

The effect of the developed optical lens on FOV and PA signal intensity was ascertained through PA imaging of tungsten wires that were placed radially; each wire with a diameter of 100 μm was positioned at -75° to 75° at 15° angular intervals, and 5 to 55 mm at 10 mm radius intervals. The wire phantom was immersed into a container filled with 3% milk solution that served as optical scatterers. For imaging, laser pulses with a length of 7 ns and a wavement of 720 nm were generated by a Nd:YAG laser excitation system (Surelite III-10, Continuum Inc., Santa Clara, CA, USA), followed by an optical parametric oscillator (Surelite OPO Plus, Continuum Inc.). The developed TRUS–PA probe was connected to a commercial US imaging system (Vantage Research Ultrasound System, Verasonics Inc., Kirkland, WA, USA), to acquire PA image data. PA images were reconstructed, using an adaptive beamforming algorithm on MATLAB (MathWorks Inc., Natick, MA, USA) [27], and these were logarithmically compressed with a dynamic range of 35 dB. Note that a laser induced the noise signals that appeared on the PA images (Figure 9) in the dynamic range. The noise can be considerably reduced when electromagnetic interference shielding methods are applied to the housing and connector of the TRUS–PA probe for the purpose of commercialization.

In visual assessment, it was seen that the TRUS–PA probe with the developed optical lens provided a higher-quality PA image than without the optical lens (Figure 9a,b). Without the developed optical lens (Figure 9a), the wires located at 38 and 48 mm barely appeared on the image because PA signal intensity was similar to the noise. Note that the distance between the probe and the front wires was about 5 mm. In addition, there were some invisible wire images even at 25 mm, which were indicated by the white arrows. When the optical lens was used, in contrast, the wire images positioned up to 35 mm were clearly observed and some wires located at 45 mm also appeared; however, the wire images on the edge were not visible. This is possible because the outer scanlines of both US and PA images were generally formed by using fewer channel datasets than the middle scanlines. For example, only 32 channel datasets are available for the outermost scanline, whereas the center scanline is formed by using 64 channel datasets. Based on the position of the wire images, it was found that the FOV of the developed TRUS–PA probe was about 120° at a depth of 25 mm, and it was 90° at 35 mm. Note that the optical lens was designed to have an FOV of 105° in the lateral-axial plane and a focal length of 25 mm for the planar–oblique lens in the elevation-axial plane. To assess the effect of the lens on PA signal intensity, the envelope signals generated from the center wires (Figure 9c) and the wires along the arc of the circle with a radius of 25 mm (Figure 9d) were obtained. The PA signals acquired with the optical lens were much higher than those without the lens (i.e., 2.98 times higher on average). From the experimental results, it could be concluded that the developed optical lens was effective in focusing irradiated laser onto the imaging plane, even in the scattering medium.

### 3.3. Combined US and PA Imaging of Targets Behind the Procine Intestine

The prostate is positioned behind the wall of the rectum. Therefore, we measured light penetration through the porcine intestine, to predict the effect of the rectal wall on the PA imaging of the prostate and to determine an optimal wavelength for PA imaging of the prostate. This experiment was necessary because some researchers have reported Monte Carlo simulation results that light intensity passing through the rectal wall is limited for transrectal PA imaging due to the high light absorption in the rectal wall. Based on the simulation results, they asserted that the transrectal approach for PA imaging of the prostate might not be suitable, and it would be difficult to achieve sufficient imaging depth, spatial resolution, and FOV for the prostate PA imaging [28,29].

**Figure 10:**
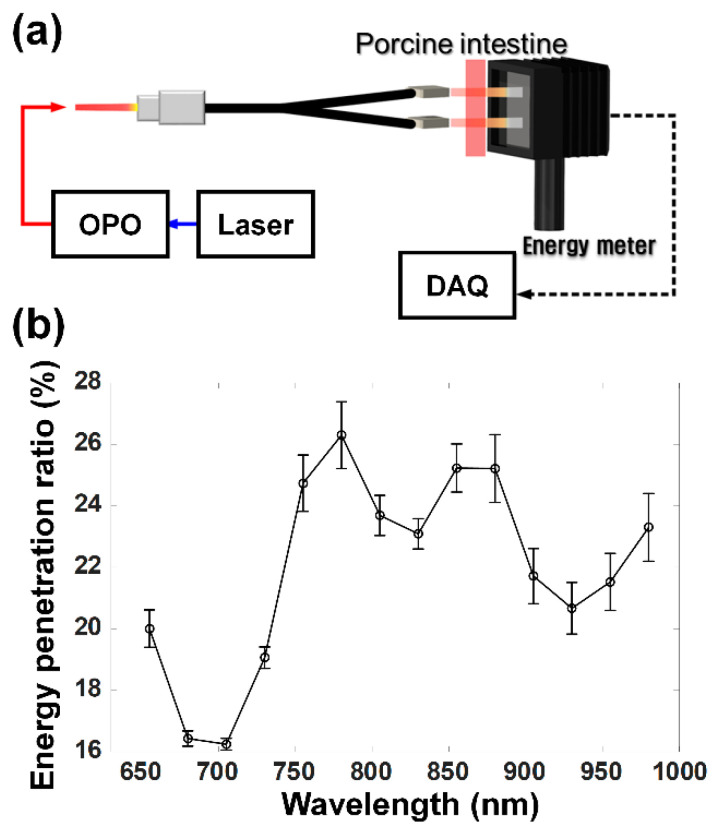
(**a**) Illustration of the experimental setup for measuring laser penetration through the porcine intestine and (**b**) ratio of laser energy penetration as a function of wavelength, which was obtained after measuring laser energy, both with and without the porcine intestine. The circles and the error bars indicate the mean and the variation. OPO and DAQ stand for optical parametric oscillator and data acquisition, respectively.

For the attenuation measurement to explore the possibility of the TRUS–PA imaging of the prostate, the Nd:YAG laser excitation system, followed by the optical parametric oscillator, was used to generate 7 nm laser pulses, as shown in Figure 10a. The laser energy delivered by the bifurcated optical fiber bundle was measured by using an energy meter (MAESTRO, Gentec-EO Inc., Quebec, QC, Canada) and recorded. The laser energy measured without the porcine intestine served as a reference at a given laser wavelength. After placing the porcine intestine between the optical fiber bundles and the energy meter, the laser passing through the porcine intestine was measured. The thickness of the porcine intestine was about 3 mm, which is similar to the median human rectal wall thickness [30]. A ratio of laser energy penetration was calculated by dividing the measured laser energy by the reference. This process was repeated by changing the wavelength from 650 to 975 nm, at 25 nm intervals. Note that the experiments were performed four times, with different porcine intestines. As shown in Figure 10b, the highest mean ratio of the laser energy penetration was 26.3% at a wavelength of 780 nm, and the average of the mean ratios at all the wavelengths was 21.9%.

**Figure 11:**
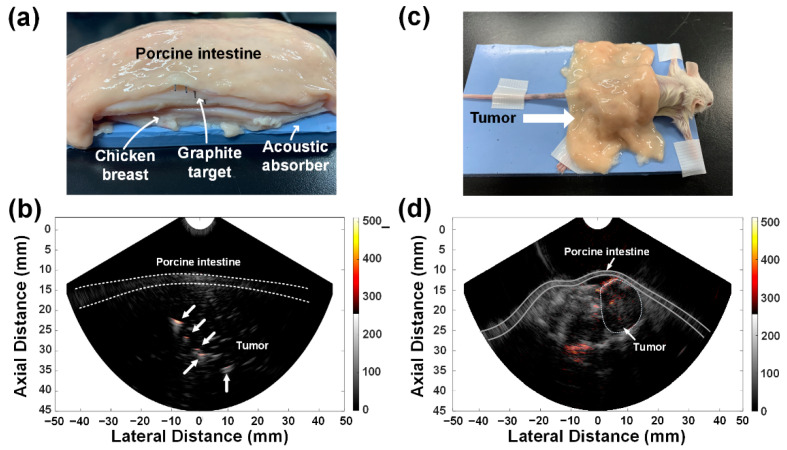
(**a**) Photograph of the imaging target used for the ex vivo experiments and (**b**) combined US and PA image of the five graphite rods in the chicken breast tissue covered by the porcine intestine. The US and PA images were logarithmically compressed with a dynamic range of 55 and 25 dB, respectively. (**c**) Photograph of the xenograft BALB/c (Albino, Immunodeficient Inbred Strain) mouse covered by the porcine intestine for the in vivo experiments and (**d**) combined US and PA images of the tumor site on the mouse. The US and PA images were logarithmically compressed with dynamic ranges of 45 and 25 dB, respectively.

**Figure A4:**
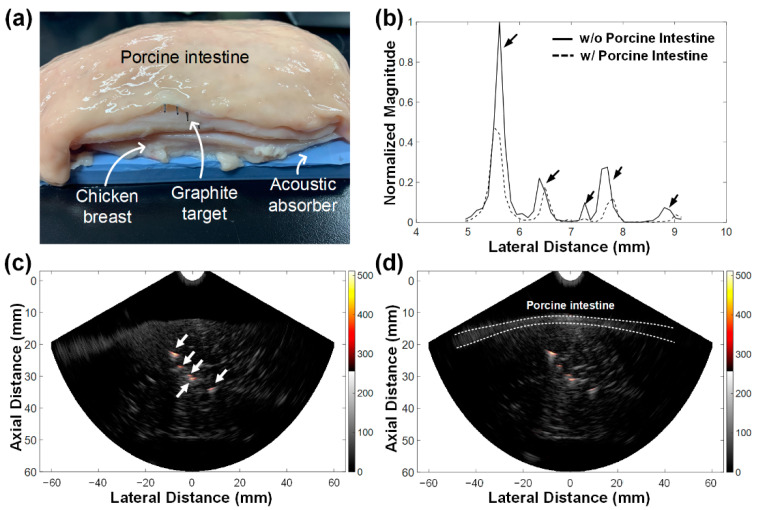
(**a**) Photograph of the imaging target used for the ex vivo experiments and (**b**) lateral beam profiles of the five graphite rods in the chicken breast tissue, without (solid line) or with (dashed line) the porcine intestine cover. (**c**,**d**) Combined ultrasound and photoacoustic images of the image target, (**c**) without and (**d**) with the porcine intestine cover. The US and PA images were logarithmically compressed with a dynamic range of 55 and 25 dB, respectively.

The feasibility of combined US and PA imaging through the porcine intestine was investigated. For this, five graphite rods with a diameter of 0.5 mm were embedded diagonally in chicken breast specimens covered by the porcine intestine, as shown in Figure 11a. For the PA imaging, a wavelength of 780 nm was selected. Despite the presence of the porcine intestine, the graphite targets were well distinguished from the speckle pattern in the US image of the chicken breast tissue, which were indicated by the white arrows in Figure 11b; the PA intensity decreased 2.4 times on average when the porcine intestine was covered, compared to that without the porcine intestine cover (see Appendix C Figure A4). Note that the measurement of the laser penetration shown in Figure 10 was conducted without the developed optical lens. Due to the beam focus on the imaging plane by the optical lens, the reduction ratio in the imaging test was smaller than the direct measurement. Unlike the previously reported simulation results, the experimental results showed the possibility of acquiring a combined US and PA image of the prostate through the human rectum intestine. The similar results were also obtained in vivo, as shown in Figure 11d.

For the in vivo experiment, the xenograft BALB/c (Albino, Immunodeficient Inbred Strain) mouse model, in which PC-3 prostate cancer cells were implanted around the thigh, was prepared. The animal experiment was conducted in accordance with the guidelines and regulations approved by the Institutional Animal Care and Use Committee of Seoul National University Bundang Hospital, South Korea. The mouse model was fixed on the acoustic absorber, and the porcine intestine was placed on the back of the mouse, enough to cover the tumor, as shown in Figure 11c. The laser wavelength was set to 780 nm for the PA imaging. Figure 11d shows the combined US and PA image of the PC-3 tumor mouse model. The white dashed line in this image represents the tumor boundary, and the two solid lines indicate the porcine intestine boundary. Note that suspicious tumors appear hypoechoic in US images [31]. The PA signals were observed around and inside the tumor, which may be evidence of the neovascularization for tumor cell growth [12,32]. Additionally, it was seen that the developed TRUS–PA probe was able to acquire the entire PA image of the mouse thigh behind the porcine intestine (i.e., about 25 mm depth from the porcine intestine), even though no contrast agent was used.

## 4. Conclusions

The primary challenge in accurate diagnosis of prostate cancer is to locate micro- and neo-vascularization accurately, as well as to delineate the cancer boundary clearly. Combined US and PA imaging is the most feasible way to achieve the goal because of high-sensitivity PA imaging of blood vessels in conjunction with US anatomic imaging; this emerging method is analogous to combined CEUS and US B-mode imaging that is less sensitive to small blood vessels and slow blood flow even if US contrast agents are used. Additionally, it is well-known that PA imaging is able to provide clear visualization of a biopsy needle. As a result, the diagnosis of prostate cancer can be another candidate for clinical application of combined US and PA imaging. This can be realized by a combined US and PA imaging system equipped with a high-performance hybrid imaging probe. Based on the ex vivo and in vivo experimental results, we believe that the FPCB, acoustic structure, and optical lens developed in this study can contribute to the realization of a high-performance TRUS–PA probe for accurate diagnosis of prostate cancer, because these features enable the developed TRUS–PA probe to improve PA image quality, as well as to have a cross-section size and a field of view comparable to those of general TRUS probes.

## Figures and Tables

**Figure A1:**
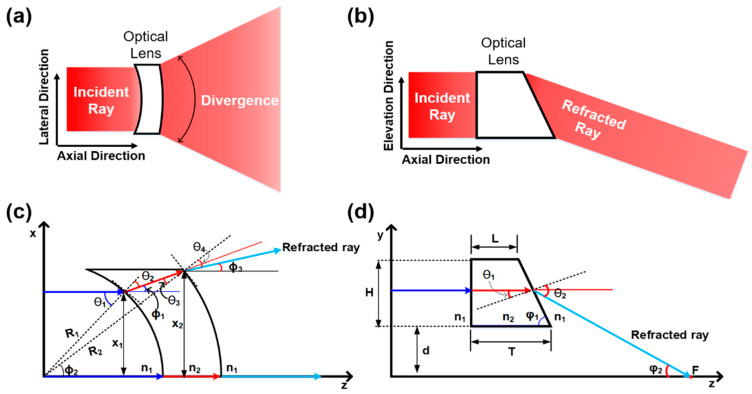
Conceptual illustration of the desired optical lens in (**a**) the lateral-axial plane and (**b**) elevation-axial plane. The models for divergence and refraction in the lens are shown in (**c**,**d**), respectively.

**Figure A2:**
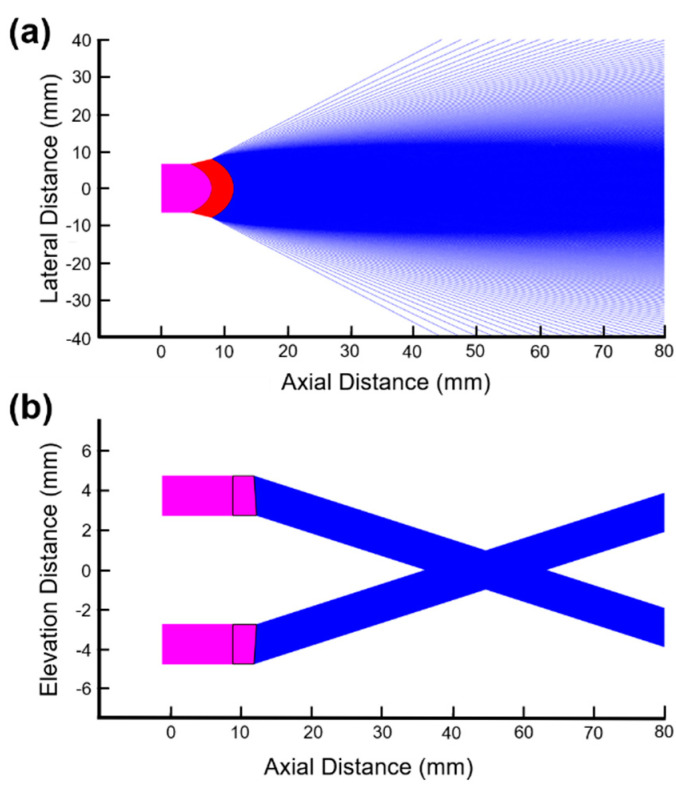
Results of numerical simulation based on the equations derived by using the ray tracing method: (**a**) concave/convex-shaped lens in the lateral-axial plane and (**b**) planar/oblique-shaped lens in the elevation-axial plane. Note that the magenta lines are collimated incoming rays, and the red lines are the refracted rays.

## Data Availability

Not applicable.

## Appendix A. Optical Lens Design

The optical lenses were designed and modeled by using the ray tracing technique [20,21]. Rays, idealized models of light, can be obtained by selecting a line that actually indicates the direction of energy flow perpendicular to light wavefront. Rays were used to model light propagation through optical systems, such as optical lenses. Ray tracing is achieved by dividing a light irradiation field into discrete rays that can be used to estimate the path of light through an optical system. Ray tracing is described by three equations, i.e., refraction, reflection, and transfer equations.

Based on the ray tracing technique, a desired optical lens could be simply designed. For the sake of simplification, we assumed that a ray was a collimated beam (i.e., light diffraction was not considered) and ignored the law of refection. The desired optical lens should produce a divergent beam in the lateral-axial plane (Appendix A Figure A1a) and a refracted beam in the elevation-axial plane (Appendix A Figure A1b); the optical lens should have a concave–convex shape in the lateral-axial plane for divergence and a planar–oblique shape in the elevation-axial plane for refraction.

For a divergent ray in the lateral-axial plane, we considered collimated rays that pass through a concave boundary. In this case, Snell’s law leads to the following:

(A1) $$sin\left(\theta_{2}\right)=\frac{n_{1}}{n_{2}}sin\left(\theta_{1}\right),$$

where $\theta_{1}$ and $\theta_{2}$ are the angles of incident and refracted rays; $n_{1}$ and $n_{2}$ are the refractive indices of air and the lens material (see Figure A1c). Therefore, $\theta_{1}$ and $\theta_{2}$ can be calculated by using the following equation:

(A2) $$\theta_{1}=sin^{-1}\left(\frac{x_{1}}{R_{1}}\right),$$

(A3) $$\theta_{2}=sin^{-1}\left(\frac{n_{1}}{n_{2}}\frac{x_{1}}{R_{1}}\right),$$

where $x_{1}$ is the radius of the incident beam, and $R_{1}$ is the radius of curvature of the concave boundary. The geometry yields are as follows:

(A4) $$\phi_{1}=\theta_{1}-\theta_{2}=sin^{-1}\left(\frac{x_{1}}{R_{1}}\right)-sin^{-1}\left(\frac{n_{1}}{n_{2}}\frac{x_{1}}{R_{1}}\right)$$

and

(A5) $$sin\left(\phi_{2}\right)=\frac{x_{2}}{R_{2}},$$

where $R_{2}$ is the radius of curvature of the convex boundary, and $x_{2}$ is determined by the following:

(A6) $$x_{2}=x_{1}+tan\left(\phi_{1}\right)\left[R_{2}cos\left(\phi_{2}\right)-R_{1}cos\left(\theta_{2}\right)\right],$$

By using Equations (A4) and (A5), at the convex boundary, $\theta_{3}$ can be expressed as follows:

(A7) $$\theta_{3}=\phi_{2}-\phi_{1}=sin^{-1}\left(\frac{x_{2}}{R_{2}}\right)-sin^{-1}\left(\frac{x_{1}}{R_{1}}\right)+sin^{-1}\left(\frac{n_{1}}{n_{2}}\frac{x_{1}}{R_{1}}\right),$$

When passing through a convex boundary, the ray also experiences Snell’s law, which is given by the following:

(A8) $$sin\left(\theta_{4}\right)=\frac{n_{2}}{n_{1}}sin\left(\theta_{3}\right)=\frac{n_{2}}{n_{1}}sin\left[sin^{-1}\left(\frac{x_{2}}{R_{2}}\right)-sin^{-1}\left(\frac{x_{1}}{R_{1}}\right)+sin^{-1}\left(\frac{n_{1}}{n_{2}}\frac{x_{1}}{R_{1}}\right)\right],$$

Finally, the divergent angle, $\phi_{3}$, can be expressed as follows:

(A9) $$\phi_{3}=\phi_{2}-\theta_{4}.$$

For the transrectal ultrasound–photoacoustic (TRUS–PA) imaging, the size of incident light (i.e., $x_{1}$) is determined by the height of an optical fiber bundle. After selecting the desired divergent angle, $\phi_{3}$, the design parameters $R_{1}$ and $R_{2}$ can be determined by using Equations (A5), (A8), and (A9). Note that if the radius of curvature of the concave boundary, $R_{1}$, is much longer than the incident beam radius, $x_{1}$, Equation (A9) can be approximately expressed as follows:

(A10) $$\phi_{3}\approx \left(1-\frac{n_{2}}{n_{1}}\right)\frac{x_{2}}{R_{2}}+\frac{n_{2}}{n_{1}}\left(1-\frac{n_{1}}{n_{2}}\right)\frac{x_{1}}{R_{1}},$$

because it is valid that sin(θ)≈θ if $\theta <14^{{}^{\circ}}$, at which an error rate is 1%.

For converging light illumination in the elevation-axial plane, collimated rays meet the planar boundary at which normal incidence occurs (see Appendix A Figure A1d). The rays are only refracted at the oblique boundary. The angle of refraction is as follows:

(A11) $$\theta_{2}\;=\;sin^{-1}\left(\frac{n_{1}}{n_{2}}sin\left(\theta_{1}\right)\right),$$

where $n_{1}$ and $n_{2}$ are the refractive indices of air and the lens material, and $\theta_{1}$ can be obtained by the following:

(A12) $$\theta_{1}=90-\varphi_{1}.$$

Note that $\varphi_{1}$ is the inclination angle of the oblique boundary. Moreover, the angle of the refracted ray to the *z*-axis is derived as follows:

(A13) $$\varphi_{2}=\varphi_{1}+\theta_{2}-90.$$

Finally, the focal length from the surface of an ultrasound transducer can be derived as follows:

(A14) $$F=L+\frac{0.5H}{tan\left(\varphi_{1}\right)}+\frac{d+0.5H}{tan\left(\varphi_{2}\right)}\;,$$

where L is the length of the short base of the planar–oblique lens, H is the height of the lens, and d is the gap between the focal depth and the lens in the elevation direction. Note that d is approximately equal to half the height of an ultrasound transducer.

## Appendix B. Numerical Simulation

The equations derived for the optical lens design cannot be expressed as a closed form. The target parameters for the concave/convex-shaped lens are the radius curvatures (i.e., $R_{1}$ and $R_{2}$), whereas the parameter for the planar/oblique-shaped lens is the inclination angle of the oblique boundary, $\varphi_{1}$. When other parameters were given, the target parameters were found by numerical simulation.

As an optical lens material, we chose Epotek-301 (Epoxy Technologies, Billerica, MA, USA) because the optical transparency of the material is 0.99. Its refractive index (i.e., $n_{2}$) is 1.519. We assumed that other media was air, of which the refractive index is 1.0003 (i.e., $n_{1}$). Since the length of the optical fiber bundle used for this study was 13 mm, the radius of the collimated beam (i.e., $x_{1}$) was set to be 6.5 mm. For efficient photoacoustic (PA) imaging of the prostate, the field of view (FOV) in the lateral-axial plane should be as wide as possible; our target FOV was wider than 100°, so the desired divergent angle, $\phi_{3}$, in Equation (A9) should be larger than 50°. With the given parameters, we conducted iterative numerical simulation to find the radius curvatures (i.e., $R_{1}$ and $R_{2}$) of the concave/convex-shaped lens. Finally, we selected a $R_{1}$ of 8 mm and a $R_{2}$ of 11.5 mm. In this case, FOV was expected to be 105° (see Appendix B Figure A2a).

For the planar/oblique-shaped lens, the height of the lens (i.e., *H*) was set to be 2 mm, the height of the optical fiber bundle was 2 mm, and the focal length (i.e., *F*) was chosen to be 25 mm. Since the height of the TRUS transducer in the elevation direction was 5.5 mm, the gap between the focal depth and the lens in the elevation direction (i.e., *d*) was set to be 2.75 mm. Moreover, the length of the short base of the planar–oblique lens *L* was 3.5 mm, which was the difference between $R_{1}$ and $R_{2}$. From the iterative numerical simulation, finally, the inclination angle of the oblique boundary (i.e., $\varphi_{1}$) was determined to be 80° (see Appendix B Figure A2b).

## Appendix C. Monte Carlo Simulation

The simulation was conducted by using a Monte Carlo light-scattering program (available from the Oregeon Medical Laser Center, https://omlc.org/software (accessed on 10 December 2020)), to ascertain the effect of optical scattering on the performance of the developed optical lens (see Appendix C Figure A3). The results imply that the direction of light scattering is determined by initial optical intensity distribution. In other words, it is not significant that the optical energy is widened by the natural diffusion, because the amount of the optical energy or energy distribution is mainly determined by the initial direction of irradiated light.
